# Book Review: Toxicogenomics in Predictive Carcinogenicity

**DOI:** 10.3389/fgene.2017.00011

**Published:** 2017-02-03

**Authors:** M. Prakash Hande

**Affiliations:** Department of Physiology, Yong Loo Lin School of Medicine, National University of SingaporeSingapore, Singapore

**Keywords:** toxicogenomics, cancer, biomarkers, cancer prediction, health risk assessment

Toxicogenomics (TGx), a discipline of toxicology allows scientists to identify and characterize the genomic signatures of environmental toxicants, as well as use gene and protein expression profiles to study the relationship between exposure and disease outcome and understand gene-environment interactions. TGx considered as the fusion of three scientific fields—toxicology, molecular biology and bioinformatics. There are numerous applications of microarray technology in environmental genomics. By monitoring thousands of genes in cells simultaneously, DNA microarray technology provides the opportunities to characterize the gene expression patterns induced by environmental toxicants. This “omics” discipline offers direct comparison of expression values for a control against an altered condition revealing a set of biomarkers indicative of that altered state. Such a biomarker or a fingerprint can eventually be used as a tool to classify toxicant exposures and to predict the mode of action of toxicant exposures. The data obtained from TGx will definitely save time and cost. TGx approaches might avoid or minimize the use of animals in toxicology studies and enable us to perform certain human studies that could not be carried out at overtly toxic exposures.

The book contains 503 pages of text and references with 14 chapters. According to the Preface, the book was conceptualized in an Applied Symposium at the 45th Annual Meeting of the Environmental Mutagenesis and Genomics Society in Orlando, FL (USA) on September 19, 2014. However, the editors subsequently decided to focus more on predicting carcinogenicity. This is an enormous task which authors have successfully exercised in completing this important book. While the effort is commendable, it may be difficult to i predict cancer based on the gene signatures *per se*, as carcinogenesis is a complex process. Nevertheless, this book provides a collection of articles in the field of TGx which would be very useful to cancer biologists and toxicologists who intend to use genomics approaches.

In Chapter 1, Michael D Waters, one of the two editors, introduces the topic of TGx and its use in predicting carcinogenicity. In addition, a thorough background on the application of –omics technologies in toxicology is also given. Several aspects of TGx are highlighted including genomics, proteomics and metabolomics. Separately, the concepts of predictive toxicology and predictive carcinogenicity are discussed using studies on different model systems. This chapter provides a good introduction to the book as well.

In subsequent chapters, following topics are discussed in a timely and succinct way.

- Genome biomarkers in cell-based drug screening (Chapter 1, HH Li).
- Gene expression signatures for differentiating genotoxic mechanisms (Chapter 2, JK Buick and CL Yauk).
- *In vivo* signatures of genotoxic and non-genotoxic chemicals (Chapter 3, SS Auerbach).
- Mode of action and risk assessment of chemicals using transcriptomic assays (Chapter 5, RS Thomas and MD Waters and 7, MM Schapp et al.).
- Evaluation of threshold in genotoxicity dose-response using transcirptomics (Chapter 6, PD McMUllen et al.).
- Embryonic stem cells as models in genotoxicity assays (Chapter 8, L. Recio).
- Cancer hazard assessment for selected chemicals – use of novel data (Chapter 9, KZ Guyton and MD Waters).
- A comprehensive review on Conazoles and cancer (Chapter 10, S Nesnow).
- Application of transcriptomics in exposed human populations (benzene study) (Chapter 11, CM McHale et al.).
- A toxicogenomics case study using Furan (Chapter 12, AF Webster, IB Lambert and CL Yauk).
- Assessment of human relevance of pathways identified by toxicogenomics data in human health risk assessment (Chapter 13, AS Kienhuis et al.).
- Bioinformatics in the assessment of cancer (Chapter 14, PR Bushel).

All chapters coherently substantiate the strength and usefulness of the data obtained from TGx studies. Though there are some overlapping concepts and details, it is a nicely assembled book. The book certainly fills the gap of not having a compilation of TGx applications and it is very much required for all toxicologists and experimental cancer biologists alike. When such technologies become easily available (cost and ease of use), they will definitely be applied in most of the toxicology experiments used in many countries. The technologies used in TGx studies such as gene expression profiling, next generation sequencing, proteomics etc. have become easily accessible in developed countries, but are still out of reach in the low- and middle-income countries. Of course, it is apparent that such studies need to be done independently in most countries as no international standards are available. This reviewer is of the opinion that no study has proven that a biomarker identified in a country for a specific chemical can be used in populations around the world. As the editors point out, full power of genomic and bioinformatics technologies has been recognized now and TGx applications have rapidly expanded since its inception. They are being used in both safety and health risk assessment. It is our hope that TGx derived gene/protein signatures predict future cancer incidences in the exposed individuals.

In my opinion, the strength of this book is the collection of individual chapters which provide valuable information on the application of TGx in toxicology and human health risk assessment. The editors deserve due credit for recruiting leading experts to write the chapters in their expertise. It would have been appropriate if each chapter contained an abstract or a summary. Redundancies on certain aspects of TGx in the chapters apart, the book is surely a collection for scientists and students in the field. Toxicologists will find this book most useful, with its comprehensive coverage of applications of TGx—*in vitro, in vivo* and epidemiology and the excellent compilation of references.

## Author contributions

The author confirms being the sole contributor of this work and approved it for publication.

### Conflict of interest statement

The author declares that the research was conducted in the absence of any commercial or financial relationships that could be construed as a potential conflict of interest.

